# Measurement properties of adult quality-of-life measurement instruments for eczema: protocol for a systematic review

**DOI:** 10.1186/s13643-015-0041-3

**Published:** 2015-04-16

**Authors:** Christian J Apfelbacher, Daniel Heinl, Cecilia A C Prinsen, Stefanie Deckert, Joanne Chalmers, Robert Ofenloch, Rosemary Humphreys, Tracey Sach, Sarah Chamlin, Jochen Schmitt

**Affiliations:** Medical Sociology, Department of Epidemiology and Preventive Medicine, University of Regensburg, 93051 Regensburg, Germany; Division of Public Health and Primary Care, Brighton and Sussex Medical School, Falmer, BN1 9PH UK; Department of Epidemiology and Biostatistics, EMGO+ Institute for Health and Care Research, VU University Medical Center, 1081 BT Amsterdam, The Netherlands; Centre for Evidence-based Healthcare, Medizinische Fakultät Carl Gustav Carus, TU Dresden, 01307 Dresden, Germany; Centre of Evidence Based Dermatology, The University of Nottingham, Nottingham, NG7 2NR UK; Department of Clinical Social Medicine, University Hospital Heidelberg, 69115 Heidelberg, Germany; Haywards Heath RH16 1UQ, West Sussex, UK; Norwich Medical School, University of East Anglia, Norwich, NR4 7TJ UK; Ann and Robert H. Lurie Children’s Hospital of Chicago, Northwestern University Feinberg School of Medicine, Chicago, IL 60611 USA

**Keywords:** Eczema, Measurement instruments, Health-related quality of life, Quality of life, Validity, Reliability, Responsiveness, Interpretability

## Abstract

**Background:**

Eczema is a common chronic or chronically relapsing skin disease that has a substantial impact on quality of life (QoL). By means of a consensus-based process, the Harmonising Outcome Measures in Eczema (HOME) initiative has identified QoL as one of the four core outcome domains to be assessed in all eczema trials (Allergy 67(9):1111-7, 2012). Various measurement instruments exist to measure QoL in adults with eczema, but there is a great variability in both content and quality (for example, reliability and validity) of the instruments used, and it is not always clear if the best instrument is being used.

Therefore, the aim of the proposed research is a comprehensive systematic assessment of the measurement properties of the existing measurement instruments that were developed and/or validated for the measurement of patient-reported QoL in adults with eczema.

**Methods/Design:**

This study is a systematic review of the measurement properties of patient-reported measures of QoL developed and/or validated for adults with eczema. Medline via PubMed and EMBASE will be searched using a selection of relevant search terms. Eligible studies will be primary empirical studies evaluating, describing, or comparing measurement properties of QoL instruments for adult patients with eczema. Eligibility assessment and data abstraction will be performed independently by two reviewers. Evidence tables will be generated for study characteristics, instrument characteristics, measurement properties, and interpretability. The quality of the measurement properties will be assessed using predefined criteria. Methodological quality of studies will be assessed using the COnsensus-based Standards for the selection of health Measurement INstruments (COSMIN) checklist. A best evidence synthesis will be undertaken if more than one study has investigated a particular measurement property.

**Discussion:**

The proposed systematic review will produce a comprehensive assessment of measurement properties of existing QoL instruments in adult patients with eczema. We aim to identify one best currently available instrument to measure QoL in eczema patients.

**Systematic review registration:**

PROSPERO CRD42015017138.

**Electronic supplementary material:**

The online version of this article (doi:10.1186/s13643-015-0041-3) contains supplementary material, which is available to authorized users.

## Background

Eczema (synonymous with atopic eczema, atopic dermatitis) is an important medical condition not only in children but also in adults. The prevalence of eczema in adults is estimated at 1% to 3% [1]. Various different interventions exist, many of which have been assessed in randomized controlled trials. Due to substantial variation in eczema outcome measures in trials, interventions are not comparable. The lack of standardization of eczema outcome measures currently renders truly evidence-based decision making difficult, if not impossible.

A multi-perspective Delphi study [2] conducted by the initiators of the Harmonising Outcome Measures in Eczema (HOME) initiative [3] defined clinical signs measured by means of a physician-assessed instrument, symptoms of eczema, and the long-term course of eczema as the core outcome domains to be applied in all future eczema trials. At the HOME II meeting in Amsterdam in 2011, the international community confirmed these core outcome measures and also added quality of life (QoL) to the core set of outcome domains [4]. The next crucial step in the process of standardizing eczema outcome measurements is to identify appropriate instruments to measure each of the four core outcome domains of atopic eczema. There was broad international consensus among clinicians, patients, and methodologists that the Outcome Measures in Rheumatology (OMERACT) quality criteria ‘Truth, Discrimination, and Feasibility’ [5] need to be met for eczema outcome measures to be recommended by the HOME initiative [4].

### Objectives

1. To systematically assess the measurement properties of patient-reported measurement instruments of QoL for adults with eczema
2. To identify outcome measurement instruments for QoL in adults with eczema
  - that meet the predefined criteria to be recommended [4,5] for the measurement of QoL in future eczema trials
  - that have the potential to be recommended in the future depending on the results of further validation studies
  - that do not meet the predefined criteria to be recommended [4,5] and therefore should not be used any more.
3. To provide the evidence base
  - for an international consensus process to further standardize the assessment of QoL in adults with eczema in clinical trials.
  - for an international consensus process to prioritize further research concerning QoL assessment in adults with eczema.

## Methods/Design

### Protocol and registration

The methods for this systematic review have been developed according to recommendations from the Preferred Reporting Items for Systematic Reviews and Meta-Analyes Protocols (PRISMA-P) statement [6]. This protocol has been registered in the International Prospective Register of Systematic Reviews (PROSPERO): CRD42015017138.

### Literature search

A systematic literature search will be performed in PubMed and EMBASE. The search strategy will contain blocks of search terms related to the following aspects:

1. construct of interest: quality of life
2. target population: (atopic) eczema (cf Table 1).

**Table 1:** **Inclusion and exclusion criteria**

	**Inclusion criteria**	**Exclusion criteria**
Population	Eczema (synonyms: atopic eczema, atopic dermatitis, neurodermatitis)	Populations with other skin diseases than eczema, populations of children with eczema, and populations of adolescents with eczema
Study design	Development study, validation study	Linguistic validation studies
Outcome	Quality of life, health-related quality of life	Signs, disease severity measure, disease control measure, biomarker, and physiology of the skin
Type of measurement instrument	Self-reported measurement instrument	All others
Publication type	Articles with available full text	Abstracts
3. measurement properties: the precise PubMed search filter for finding studies on measurement properties developed by Terwee *et al*. will be used to identify relevant articles [7]. This filter has a sensitivity of 93.1% and a precision of 9.4%.
4. interpretability

The entire search strategy is available as an Additional file 1 to this protocol.

The systematic electronic search will be supplemented by hand searching of reference lists of studies included and key articles on this topic. Furthermore, an additional search will be performed in each database, including the names of the instruments which are found in the initial search. The PROQOLID (www.proqolid.org) database will be searched.

### Eligible measurement instruments

Eligible measurement instruments will include all patient-reported measurement instruments which were designed and/or validated to measure QoL in adults with eczema.

### Eligible studies

A study will be included if it is published as a full-text paper and concerns the development (‘development paper’) and/or evaluation of the measurement properties (‘validation paper’) of instruments that measure QoL or health-related quality of life (HrQoL) in adult people with eczema. A study with a mixed patient sample will be eligible either if it presents a subgroup analysis for adult patients with atopic eczema or if adult patients with atopic eczema constitute at least 50% of the study population. The measurement instrument must be a self-reported questionnaire. Articles that report indirect evidence, for instance, by using data obtained within the context of a clinical trial, will not be considered eligible. Articles assessing the measurement properties of dermatology-specific instruments in non-eczema samples will not be considered eligible.

### Study selection

Two reviewers will independently judge titles and abstracts retrieved in the literature search and, at a second stage, full-text articles for eligibility (Table 1). Disagreements will be resolved by discussion with all reviewers.

### Assessment of the methodological quality of included studies

The COnsensus-based Standards for the selection of health Measurement Instruments (COSMIN) checklist [8-10] will be used to evaluate the methodological quality of included studies. In the COSMIN checklist (cf www.cosmin.nl), four domains are distinguished (reliability, validity, responsiveness, and interpretability) with related measurement properties and aspects of measurement properties. These are listed in Table 2 (adapted from [8]).

**Table 2:** **Definitions of domains, measurement properties, and aspects of measurement properties**

**Domain**	**Measurement property**	**Aspect of a measurement property**	**Definition**
Reliability			The degree to which the measurement is free from measurement error
Reliability (extended definition)			The extent to which scores for patients who have not changed are the same for repeated measurement under several conditions: for example, using different sets of items from the same HR-PROs (internal consistency) over time (test-retest) by different persons on the same occasion (inter-rater) or by the same persons (that is, raters or responders) on different occasions (intra-rater)
	Internal consistency		The degree of interrelatedness among the items
	Reliability		The proportion of total variance in the measurements which is because of ‘true’^a^ differences among patients
	Measurement error		The systematic and random error of a patient’s score that is not attributed to true change of the construct to be measured
Validity			The degree to which an HR-PRO instrument measures the construct(s) it purports to measure
	Content validity		The degree to which the content of an HR-PRO instrument is an adequate reflection of the construct to be measured
		Face validity	The degree to which (the items of) an HR-PRO instrument indeed looks as though they are an adequate reflection of the construct to be measured
	Construct validity		The degree to which the scores of an HR-PRO instrument are consistent with hypotheses (for instance with regard to internal relationships, relationships to scores of other instruments, or differences between relevant groups) based on the assumption that the HR-PRO instrument validly measures the construct to be measured
		Structural validity	The degree to which the scores of an HR-PRO instrument are an adequate reflection of the dimensionality of the construct to be measured
		Hypothesis testing	Idem construct validity
		Cross-cultural validity	The degree to which the performance of the items on a translated or culturally adapted HR-PRO instrument is an adequate reflection of the performance of the items of the original version of the HR-PRO instrument
Responsiveness			The ability of an HR-PRO instrument to detect change over time in the construct to be measured
	Responsiveness		Idem responsiveness
Interpretability^b^			The degree to which one can assign qualitative meaning - that is, clinical or commonly understood connotations - to an instrument’s quantitative scores or changes in scores

*Abbreviations*: *HR-PROs* health related patient-reported outcomes, *CTT* classical test theory. ^a^The word ‘true’ must be seen in the context of the CTT, which states that any observation is composed of two components - a true score and error associated with the observation. ‘True’ is the average score that would be obtained if the scale were given an infinite number of times. It refers only to the consistency of the score and not to its accuracy [14]. ^b^Interpretability is not considered a measurement property but an important characteristic of a measurement instrument.

For each of the measurement properties, the COSMIN checklist consists of 5 to 18 items covering methodological standards (organized in nine boxes for the nine measurement properties). In addition, each item can be scored on a four-point rating scale (that is, ‘poor,’ ‘fair,’ ‘good,’ ‘excellent’). Taking the lowest rating for each item in one box, an overall quality score (‘poor,’ ‘fair,’ ‘good,’ ‘excellent’) is obtained for each measurement property separately.

The measurement property ‘criterion validity’ will not be considered for the purpose of this systematic review since no gold standard exists for QoL.

### Data abstraction

Relevant data from all included articles will be summarized in evidence tables. The evidence table will be drafted and pilot tested. Data from each article included will be abstracted independently by two reviewers. All reviewers will participate in this process and will work in pairs on defined sets of articles. Disagreements will be resolved by discussion of all reviewers.

Evidence tables will include the following: reference, geographical location, language, setting, study type, key characteristics of study subjects, name of measure, domains measured, number of items and (sub)scales, number and type of response categories, recall period in the questions, scoring algorithm, time needed for administration, mode of administration, target population for whom the questionnaire was originally developed, how a full copy of the questionnaire can be obtained, the instructions given to those who complete the questionnaire, the available versions and translations of the questionnaire, results of the measurement properties, all items from the COSMIN box Generalisability, and all items from the COSMIN box Interpretability [8,9].

If general characteristics of an instrument (that is, name of measure, number of items and (sub)scales, number and type of response categories, recall period in the questions, scoring algorithm, time needed for administration, mode of administration, target population for whom the questionnaire was originally developed, how a full copy of the questionnaire can be obtained, the instructions given to those who complete the questionnaire, the available versions and translations of the questionnaire) cannot be extracted from the studies included, the original development paper may be consulted to obtain missing information.

### Content comparison

An overview of the content of each instrument on item level will be presented in order to visualize which content is covered by the different instruments. The original development paper is going to be consulted to obtain this information.

### Quality assessment of the measurement instruments

The predefined criteria for rating the quality of measure recommended by the COSMIN group will be used [11] (cf Table 3). These criteria are in accordance with the OMERACT filter [5] which has been adopted by the HOME initiative [4] and the criteria applied in a previous review on atopic eczema outcome measures [12] (Table 3).

**Table 3:** **Quality criteria for measurement properties adapted from** [11] **and** [15]

**Property**	**Rating**	**Quality criteria**
Reliability		
Internal consistency	+	Cronbach’s alpha(s) ≥ 0.70
	?	Dimensionality not known OR Cronbach’s alpha not determined
	−	Cronbach’s alpha(s) < 0.70
	+	MIC > SDC OR MIC outside the LOA
	?	MIC not defined
	−	MIC ≤ SDC OR MIC equals or inside LOA
Reliability	+	ICC/weighted Kappa ≥ 0.70, OR Pearson’s *r* ≥ 0.80
	?	Neither ICC/weighted Kappa nor Pearson’s *r* determined
	−	ICC/weighted Kappa < 0.70 OR Pearson’s *r* < 0.80
Validity		
Content validity	+	All items are considered to be relevant for the construct to be measured, for the target population, and for the purpose of the measurement, AND the questionnaire is considered to be comprehensive
	?	Not enough information available
	−	Not all items are considered to be relevant for the construct to be measured, for the target population, and for the purpose of the measurement, OR the questionnaire is considered not to be comprehensive
Construct validity		
Structural validity	+	Factors should explain at least 50% of the variance
	?	Explained variance not mentioned
	−	Factors explain <50% of the variance
Structural validity (IRT methods applied)	+	Residual correlations among the items after controlling for the dominant factor <0.20 OR Q3′s < 0.37, item scalability >0.30, IRT model fit: G2 > 0.01, no DIF for important subject characteristics (such as age, gender, education): McFadden’s R2 < 0.02
	?	Important characteristics not reported
	−	Residual correlations among the items after controlling for the dominant factor ≥0.20 OR Q3′s ≥ 0.37, item scalability ≤0.30, IRT model fit: G2 ≤ 0.01, important DIF for important subject characteristics (such as age, gender, education): McFadden’s R2 ≥ 0.02
Hypothesis testing	+	Correlation with an instrument measuring the same construct ≥0.50 OR at least 75% of the results are in accordance with the hypotheses, AND correlation with related constructs is higher than with unrelated constructs
	?	Solely correlations determined with unrelated constructs
	−	Correlation with an instrument measuring the same construct <0.50 OR <75% of the results are in accordance with the hypotheses OR correlation with related constructs is lower than with unrelated constructs
Cross-cultural validity	+	No differences in factor structure OR no important DIF between language versions
	?	Multiple group factor analysis not applied AND DIF not assessed
	−	Differences in factor structure OR important DIF between language versions
Responsiveness		
Responsiveness	+	Correlation with changes on instruments measuring the same construct ≥0.50 OR at least 75% of the results are in accordance with the hypotheses OR AUC ≥ 0.70, AND correlations with changes in related constructs are higher than with unrelated constructs
	?	Solely correlations determined with unrelated constructs
	−	Correlations with changes on instruments measuring the same construct <0.50, OR <75% of the results are in accordance with the hypotheses, OR AUC < 0.70, OR correlations with changes in related constructs are lower than with unrelated constructs
Interpretability	+	MIC calculated and anchor questions clearly described
	?	MIC calculated but anchor questions not clearly labelled
	−	MIC not reported

MIC: minimal important change, SDC: smallest detectable change, LOA: limits of agreement, ICC: intraclass correlation coefficient, AUC: area under the curve. +positive rating, ? indeterminate rating, −negative rating.

### Best evidence synthesis

If an instrument has been evaluated in different studies, findings will be synthesized if the characteristics of the included studies are sufficiently similar and if the results of the studies do not show too different or conflicting findings and if the methodological quality of the included studies is sufficient [13]. The criteria for best evidence synthesis are outlined in Table 4.

**Table 4:** **Levels of evidence for the overall quality of a measurement property** [16]

**Level**	**Rating**	**Criteria**
Strong	+++ or −−−	Consistent findings in multiple studies of good methodological quality OR in one study of excellent methodological quality
Moderate	++ or −−	Consistent findings in multiple studies of fair methodological quality OR in one study of good methodological quality
Limited	+ or −	One study of fair methodological quality
Conflicting	+/−	Conflicting findings
Unknown	?	Only studies of poor methodological quality

+positive rating, ? indeterminate rating, −negative rating.

### Generating recommendations for the use of QoL measurement instruments for eczema

For each instrument identified in the review, a standardized recommendation for usage or required future validation work will be made depending on the quality of the instrument and on the methodological quality of included studies (cf Table 5). According to the results of the HOME II meeting [4], all three criteria of the OMERACT filter [5], that is, truth, discrimination, and feasibility, have to be met by an outcome measure to be recommended by the HOME initiative.

**Table 5:** **Quality criteria required for recommendation of QoL measures for eczema**

**Quality item (name)**	**Inclusion in OMERACT filter**	**Required rating for recommendation**
Content validity	Truth	+
Structural validity	Truth	+
Hypotheses testing	Truth	+
Cross-cultural validity	Truth	+
Internal consistency	Discrimination	+
Reliability	Discrimination	+
Measurement error	Discrimination	+
Responsiveness	Discrimination	+
Interpretability	Feasibility	+

Four categories of recommendation will be made:

1. QoL measurement instrument meets all requirements and is recommended for use.
2. QoL measure meets two or more quality items, but performance in all other required quality items is unclear, so that the outcome measure has the potential to be recommended in the future depending on the results of further validation studies.
3. QoL measure has low quality in at least one required quality criterion (≥1 rating of ‘minus’) and therefore is not recommended to be used any more.
4. QoL measure has (almost) not been validated. Its performance in all or most relevant quality items is unclear so that it is not recommended to be used until further validation studies clarify its quality.

Finally, we aim to identify one best (currently available) instrument to assess QoL in adult eczema.

## Discussion

The proposed systematic review will yield a comprehensive assessment of measurement properties of existing QoL instruments in adult patients with eczema. We aim to arrive at a recommendation of one best instrument to measure QoL in eczema patients. The processes underlying this systematic review are transparent and systematic. Quality assurance is achieved by involving two independent reviewers at each stage. A strength of the proposed research is the international coverage of the contributing reviewers. This will increase the credibility of any findings. However, coordinating work packages between many reviewers is certainly a challenge. Whether or not we will be able to reach the goal of recommending one best instrument is unclear. It may well be that no instrument will meet all the filter criteria or that several instruments will meet them. In any case, the findings of this systematic review will inform a consensus-finding process at the fourth meeting of the HOME initiative (HOME IV) that will take place in Malmö, Sweden, in April 2015. Based on the findings of this work, we hope to be able to inform group discussion and consensus voting with the ultimate goal to endorse one instrument to be included in the core set of outcome measurement instruments for eczema. If instruments lack important requirements, for instance, in relation to responsiveness or feasibility, further validation work will need to be done before a QoL instrument can be included in the core set.

## Additional file

Additional file 1:
**Search strings.** The search strings for Medline (via PubMed) and EMBASE.

## Abbreviations

*COSMIN*: COnsensus-based Standards for the selection of health Measurement INstruments
*HrQoL*: health-related quality of life
*PRO*: patient-reported outcome
QoL: quality of life
